# Electrocarboxylation: towards sustainable and efficient synthesis of valuable carboxylic acids

**DOI:** 10.3762/bjoc.10.260

**Published:** 2014-10-27

**Authors:** Roman Matthessen, Jan Fransaer, Koen Binnemans, Dirk E De Vos

**Affiliations:** 1 Centre for Surface Chemistry and Catalysis, KU Leuven, Arenbergpark 23, B-3001 Leuven, Belgium; 2 Department of Metallurgy and Materials Engineering, KU Leuven, Arenbergpark 44, B-3001 Leuven, Belgium; 3 Department of Chemistry, KU Leuven, Celestijnenlaan 200F, B-3001 Leuven, Belgium

**Keywords:** carbon dioxide, carboxylic acids, counter electrode reaction, electrocarboxylation mechanism, reactor setup

## Abstract

The near-unlimited availability of CO_2_ has stimulated a growing research effort in creating value-added products from this greenhouse gas. This paper presents the trends on the most important methods used in the electrochemical synthesis of carboxylic acids from carbon dioxide. An overview is given of different substrate groups which form carboxylic acids upon CO_2_ fixation, including mechanistic considerations. While most work focuses on the electrocarboxylation of substrates with sacrificial anodes, this review considers the possibilities and challenges of implementing other synthetic methodologies. In view of potential industrial application, the choice of reactor setup, electrode type and reaction pathway has a large influence on the sustainability and efficiency of the process.

## Introduction

### Carbon dioxide recycling

**Scheme 1:**
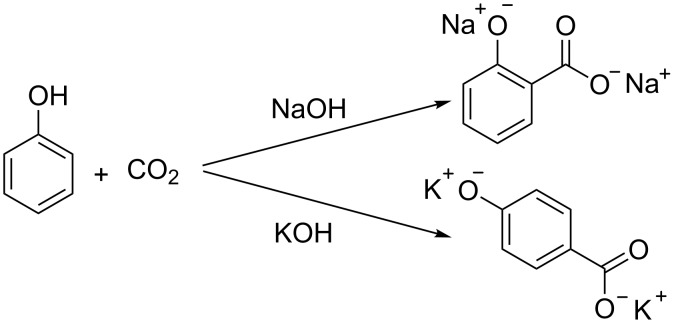
Synthesis of salicylic acid and *p*-hydroxybenzoic acid via Kolbe–Schmidt reaction [16–20].

Implementing sustainable, resource-efficient chemical processes to meet the world’s growing demand for energy and chemicals is one of today’s major challenges. Depletion of fossil resources and the ongoing increase of atmospheric carbon dioxide levels urge to investigate alternative pathways to close the carbon cycle. The use of carbon dioxide as chemical feedstock is a logical strategy for this purpose, creating economical benefit from its capture. At the moment, only a very minor fraction (<1%) of anthropogenic CO_2_ emissions is actually used [1]. As an end product of combustion, CO_2_ has a high thermodynamic stability (Δ*G*_f_° = –396 kJ/mol), often demanding for an energy intensive activation. The hydrogenation of CO_2_ to methane for example is an industrial process which requires high temperatures and pressures to activate CO_2_ [2–3]. Efficient chemical incorporation of CO_2_ is limited to rather reactive substrates, like epoxides [4–6] and amines [7–9] to produce cyclic carbonates and carbamates, respectively, and even then, elevated reaction temperatures and/or complex catalyst systems are sometimes required. Urea is the main industrial product for which CO_2_ is applied as a C_1_ building block in a reaction with ammonia, still requiring pressures around 200 bar to drive the equilibrium to acceptable yields [10–11]. Another important end product of CO_2_ are inorganic carbonates, like CaCO_3_, produced by fast reaction with metal hydroxides [12]. Carboxylic acids are an interesting class of products, as important intermediates in the synthesis of polymers and pharmaceuticals. Hydroxybenzoic acids are among the few chemicals that are industrially produced from CO_2_, via the Kolbe–Schmidt reaction at high temperatures and CO_2_ pressures [13–15]. Sodium phenolate is selectively converted to salicylic acid, a precursor of Aspirin, while potassium phenolate exclusively yields *p*-hydroxybenzoic acid, used in polyester synthesis (Scheme 1) [16–20].

**Scheme 2:**
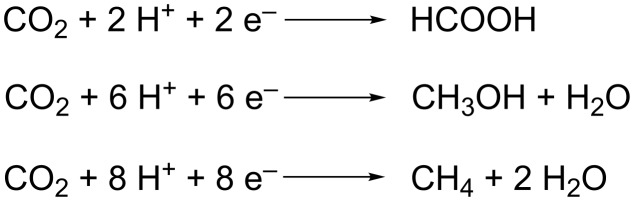
Electroreduction of carbon dioxide to formic acid, methanol or methane.

In order to generate carboxylic acids from CO_2_ under relatively mild conditions, reactive organometallic nucleophiles such as Grignard reagents can be used, generating a large amount of waste [21–24]. Electroreduction of CO_2_ can be a worthy alternative for these dangerous energy-intensive processes, replacing toxic or hazardous reducing agents by clean electrons. In this case, the high thermodynamic stability of CO_2_ is by-passed by a simple one-electron reduction at an electrode, leading to in situ generation of reactive intermediates. Often, room temperature conditions are sufficient, considering that the energy of the electrons is determined by the applied voltage [25]. Since the electroreduction takes place on a cathode surface, the need for complex homogeneous organometallic catalysts is minimized. Furthermore, electricity will be increasingly of renewable origin in the future, making organic electrosynthesis a promising technology for environmentally friendly chemical processes [26]. The electroreduction of CO_2_ can be applied for the synthesis of fuels like formic acid [27], methanol [28] or methane [29] via two-, six- and eight-electron reductions, respectively (Scheme 2). This way electric energy from periodic sustainable origin, like solar or wind energy, can be stored [30].

## Review

**Scheme 3:**
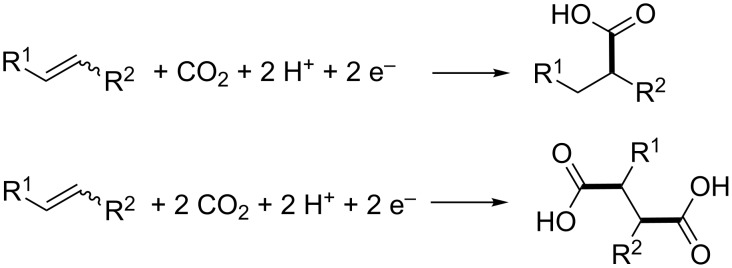
Electrochemical fixation of CO_2_ in olefins.

In this review, the focus will be on another approach, in which CO_2_ is fixed in organic chemicals by means of an energy-efficient reduction process to produce valuable carboxylic acids. This methodology requires only one or two electrons per CO_2_ molecule, as shown in Scheme 3 for olefins, with the C–C bond formation highlighted in bold.

### Industrial organic electrosynthesis

**Scheme 4:**
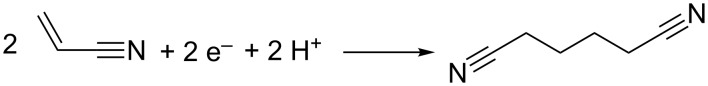
Electrohydrodimerisation of acrylonitrile to adiponitrile [32].

The chemical industry is devoting increasing research efforts to the field of organic electrosynthesis [31]. An extended series of electro-organic processes have already been implemented on an industrial scale, like for example the electrohydrodimerisation of acrylonitrile (Scheme 4) [32], or the production of *p*-methoxybenzaldehyde [33].

**Scheme 5:**
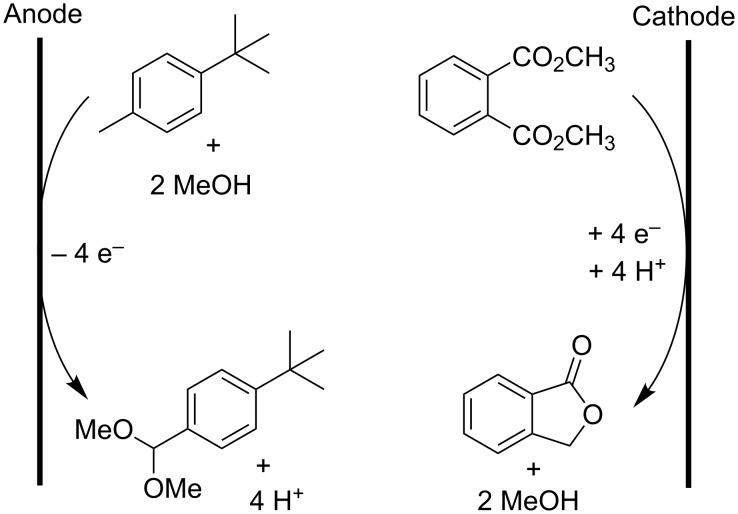
Parallel paired electrosynthesis of phthalide and *tert*-butylbenzaldehyde dimethylacetal [34].

Another interesting industrial process is the simultaneous production of phthalide and *tert*-butylbenzaldehyde dimethylacetal from dimethyl phthalate and *tert*-butyltoluene, respectively (Scheme 5). After separation, by distillation and precipitation, both products can be used in the production of pesticides [34]. This is an example of a paired electrosynthesis, in which the anodic and cathodic reactions simultaneously form compounds that are valuable. This way a combined electrochemical yield, i.e., the fraction of supplied current going to the desired reaction, is achieved, reducing energy consumption and reaction time. This methodology is very environmentally friendly since there is no generation of toxic wastes, electrical current is used more efficiently and a high atom economy is achievable [35]. The latter being the fraction of the molecular mass of all reactants which is transferred to the desired product(s). In Scheme 5, the total atom efficiency is 100% and the electrochemical yield reaches 180% [34].

Moreover, numerous pilot scale processes have been demonstrated like the electrohydrodimerization of formaldehyde to ethylene glycol [36] or the production of glyoxylic acid [37]. The most important reasons for this raised interest are the higher energy efficiency compared to traditional thermochemical processes, the use of less expensive starting materials, less aggressive reaction conditions, fewer processing steps and the discovery of unique synthesis routes [31].

Despite numerous publications and patents in the field of CO_2_ electroreduction, no industrial processes are known in which CO_2_ is electrochemically incorporated in organic chemicals producing carboxylic acids. This review will give an overview of various types of electrocarboxylation procedures bearing in mind the requirements for future industrial application. Besides the identification of a profitable market for the product, minimized process costs are the major requirement for potential large scale implementation. Some parameters that influence these costs are current efficiency, reactor design, electrode material and reactant costs.

### Electrocarboxylation setups

**Scheme 6:**
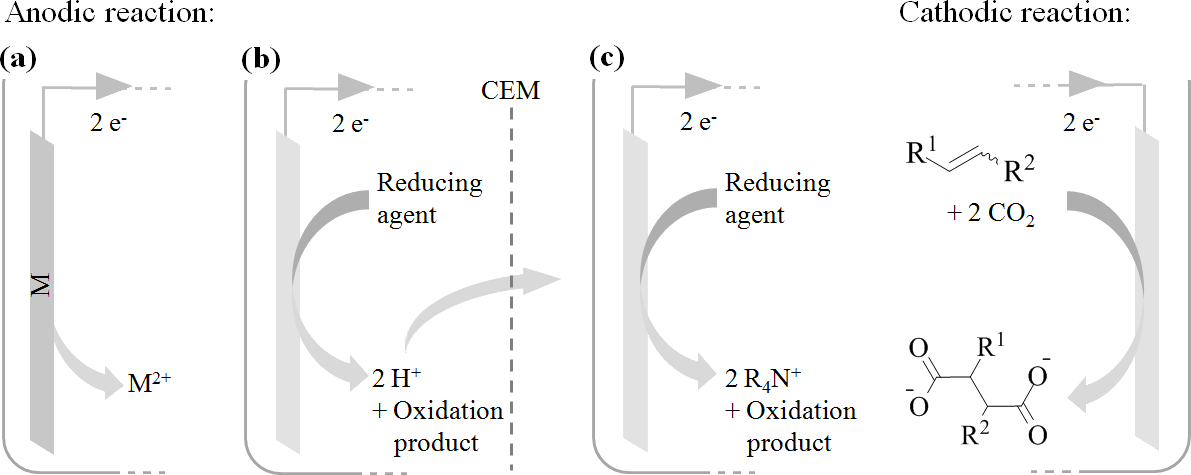
Overview of electrocarboxylation setups using (a) a sacrificial anode, (b) an inert anode, generating protons, with a cation exchange membrane (CEM) and (c) an inert anode, releasing tetraalkylammonium cations.

All electrochemical processes involve an anodic and cathodic reaction in order to close the electron cycle. Electrons supplied at the cathode must emerge from an oxidation reaction occurring at the anode (Scheme 6). The electrochemical system should be optimized to prevent unfavorable interference between both reactions, since such conflicts cause electric current to be lost and lead to a decrease in faradaic efficiency. Electrocarboxylation, the electrochemical fixation of carbon dioxide in organic chemicals, involves the electroreduction of carbon dioxide and/or an organic substrate. For olefins, alkynes, carbonyl compounds, imines and organic halides, this leads to the formation of carboxylate anions. A counter cation is required in order to obtain a stable reaction product and an anodic reaction is necessary to complete the electron cycle. The anodic generation of this counter cation has been a challenging and important point of discussion for many years, as will become clear further in this review. An overview of different possible setups for obtaining carboxylic acids is given in Scheme 6.

Electrocarboxylation reactions can either be conducted with a sacrificial anode, like magnesium or aluminum, or with an inert anode, like platinum or carbon. Most research has been focused on the fixation of CO_2_ using sacrificial anodes [38–40] (Scheme 6a). The higher oxidation potential of sacrificial anodes compared to that of the other reaction species makes this setup readily compatible with a simple undivided electrolysis cell, without a membrane separating the catholyte from the anolyte. This way high current densities can be obtained at relatively low potentials leading to minimized energy consumption. The absence of unwanted anodic reactions allows maintaining high current efficiencies without real difficulty. Furthermore, this counter electrode reaction delivers metal cations (Mg^2+^, Al^3+^), which rapidly are coordinated by the carboxylate anions formed at the cathode. Finally, the corresponding metal carboxylates can be precipitated from organic solvents allowing easy product isolation. Alongside all the benefits that are associated with these sacrificial anodes, the gradual consumption of the anode material is a major drawback for industrial applications. Not only is it rather expensive to consume such large amounts of metal; it also strongly hinders the implementation of a continuous process. Additionally, in order to obtain the free acids, an acid hydrolysis step is required, complicating product purification, and generating a significant amount of waste. The potential industrial use of electrochemical CO_2_ fixation with sacrificial anodes should be found in fine chemical applications, preferably when the carboxylate salt can be used as such.

Considerable efforts have been made in investigating other electrocarboxylation systems. Replacing the sacrificial anode with a stable anode brings along several challenges. First of all, a counter electrode reaction must be identified, delivering counter cations to balance the charge of the carboxylate anions. The anodic reactant should be more easily oxidized than the other species present in the reaction mixture. The possible side product formed in this anodic reaction should either have no effect on the system, or be a useful reactant for the cathodic reaction. The direct formation of free carboxylic acids is a very interesting approach in this respect, minimizing the amount of process steps and waste (Scheme 6b). Protons produced at the anode, however, can have a detrimental effect on the electrocarboxylation efficiency, through cathodic formation of hydrogen, formic acid and other side products [41–42]. Hydrogen formation can be limited by usage of cathode materials with high hydrogen overvoltage like lead and mercury, or more environmentally friendly tantalum and zinc [43]. In order to minimize other side reactions a cation exchange membrane (CEM) is necessary, allowing different conditions in both compartments, giving a controlled supply of protons to the catholyte. Besides the implementation and maintenance costs of such a membrane, it also causes an elevated ohmic resistance between the electrodes decreasing the energy efficiency of the process. Moreover, most membranes have difficulty operating in organic solvents and under high pressure conditions, limiting operational conditions [44]. The anodic oxidation of more reduction stable tetraalkylammonium salts is another approach compatible with non-sacrificial anodes (Scheme 6c). Here, the released tetraalkylammonium cations function as counter ions for the cathodically formed carboxylate anions. If the corresponding oxidation products are not harmful for the cathodic reaction a simple undivided cell can be envisaged. However, this can also be considered as a sacrificial process, since the tetraalkylammonium salts are consumed during the reaction. But more importantly, in contrast to the use of a dissolving anode, this method allows a more efficient implementation of a continuous process.

The first reports on electrocarboxylation date back to the early 1960s with a patent of Loveland, demonstrating the dicarboxylation of 1,3-butadiene in a two-compartment cell with a mercury cathode and a platinum anode, giving a mixture of mono- and dicarboxylic acids. CO_2_ bubbling through a catholyte solution of 1 wt % water in DMF, saturated with butadiene, yielded up to 50% of 3-hexenedioic acid [45], a result which unfortunately was difficult to reproduce [42,46]. At that time, various substrates, like olefins, alkynes and aromatic ketones, have been electrocarboxylated in these divided cells [47]. Shortly after, sacrificial anodes made the use of a diaphragm obsolete, giving satisfactory product yields and current efficiencies in an undivided cell [38]. From this point on most of the research in the field of CO_2_ fixation was focused on this type of setup.

### Electrocarboxylation of conjugated dienes

**Scheme 7:**
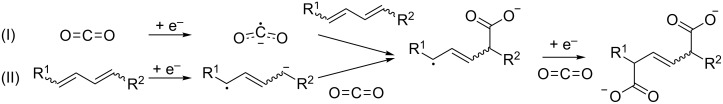
General mechanism of the electrochemical dicarboxylation of conjugated dienes [49].

The electrochemical fixation of carbon dioxide in 1,3-butadiene has been extensively investigated because of the importance of adipic acid for the polymer industry. 1,3-Butadiene is widely available, not only from steam cracking, but increasingly from dehydrogenation of linear butenes. Dicarboxylation of 1,3-butadiene yields a mixture of 3-hexene-1,6-dioic acid isomers, which are only one hydrogenation step removed from adipic acid, a monomer of nylon [41–42,44,48–49]. The general mechanism of CO_2_ fixation in conjugated dienes is illustrated in Scheme 7. It is remarkable that only one electron is used per CO_2_ molecule which is incorporated, making this a very energy efficient approach. There are two possible pathways to reach the monocarboxylate radical anion intermediate: one in which first CO_2_ is reduced to a reactive CO_2_^•−^ radical anion, and another one in which a radical anion is formed from the alkene. It has been illustrated that both pathways may be operative at the same time [49–50]. Which mechanism is favored depends amongst other parameters mainly on the diene type [49–51], CO_2_ pressure [50] and cathode material [50].

The different reactor setups used for the electrocarboxylation of 1,3-butadiene and the effect of the counter cation are illustrated in Table 1.

**Table 1:** Electrocarboxylation of 1,3-butadiene in different setups.^a^

Entry	Anode	Reducing agent	CEM^b^	Solvent (catholyte – anolyte)	C5:C6:C10^c^	η^d^ (%) (Yield (%))	Ref.

1^e^	Pt	H_2_O	Yes	CH_3_CN – H_2_O^f^	67:0:33	18 (11)	[41]
2^g^	Pt	dry MgO	Yes	CH_3_CN – CH_3_CN	26:58:16	31 (21)	[41]
3^g^	Pt^h^	H_2_	No	CH_3_CN	0:0:0	0 (0)	[42]
4^g^	Pt^h^	H_2_ + dry MgO	No	CH_3_CN	75:25:0	3.7 (–)	[42]
5^g,i^	Pt^h^	H_2_O	Yes	DMF – H_2_O^f^	–:–:–	3.4 (–)	[42]
6^g^	Pt	NH_3_	No	CH_3_CN	33:54:13	5.8 (–)	[42]
7^j^	Pt	(TEA)_2_oxalate + TEA formate^k^	No	CH_3_CN	36:52:12	39 (98)	[44]
8^l^	Mg	Anode	No	DMF	2:98:0	– (81)	[48]
9^m^	Al	Anode	No	DMF	0:100:0	42 (84)	[49]

^a^Reactions were performed in conditions presented in corresponding references; ^b^cation exchange membrane; ^c^C5:C6:C10 = 3-pentenoic acid:3-hexenedioic acid:3,7-decadienedioic acid; ^d^total current efficiency; ^e^mercury cathode; ^f^1 wt % H_2_SO_4_ solution; ^g^lead cathode; ^h^platinum hydrogen gas-diffusion electrode; ^i^NH_3_ present in catholyte; ^j^carbon felt cathode; ^k^TEA = tetraethylammonium; ^l^tantalum cathode with 2,4,4-trimethyl-1,5,9-triazacyclododecene nickel(II) tetrafluoroborate mediator; ^m^nickel cathode.

**Scheme 8:**
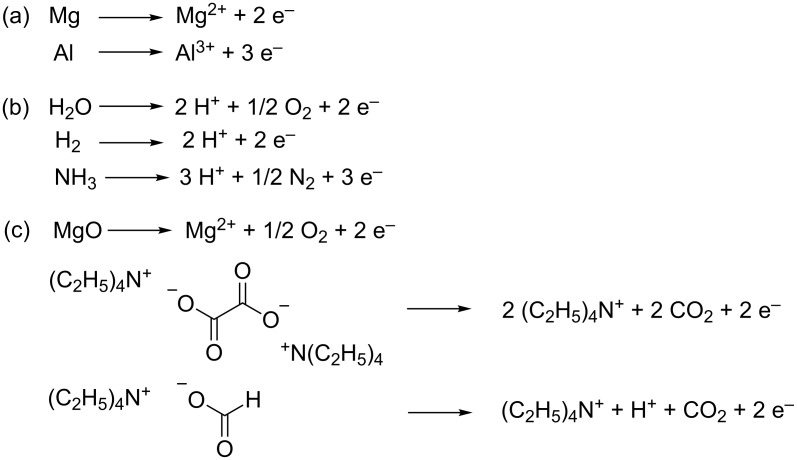
Reported anodic reactions for the electrocarboxylation of 1,3-butadiene.

The different anodic reactions that have been used for the electrocarboxylation of 1,3-butadiene (Table 1) are illustrated in Scheme 8. They are divided into three categories, with (a) the sacrificial anode dissolution, (b) the proton forming reactions and (c) the oxidations evolving other free cations.

In case a stable platinum anode is used, a C5:C6:C10 product mixture is formed, containing isomers of 3-pentenoic acid (C5), 3-hexenedioic acid (C6) and 3,7-decadienedioic acid (C10) (Table 1, entries 1–7). The product distribution is among other things influenced by the water and proton content in the reaction system. In a divided cell, in which the anolyte consists of 1% H_2_SO_4_ in water, no C6 product is formed (Table 1, entry 1). Only in an anhydrous catholyte and anolyte, applying MgO in the anolyte as reducing agent, the carboxylation becomes more selective for C6, reaching appreciable current efficiencies (Table 1, entry 2). Furthermore, the presence of water in the catholyte decreases the total current efficiency, due to the formation of formic acid [41–42]. It must be noted that, when using an aqueous and organic solvent in anolyte and catholyte, respectively, the transfer of hydrated protons through the membrane will cause water to enter the catholyte. Protons in turn can also favor formic acid generation, but on top of that, they promote the C5 and C10 formation [41]. It must be noted that the presence of formic acid illustrates the existence of mechanism I (Scheme 7). When working in an undivided cell and using protons as sole counter cation through hydrogen oxidation in anhydrous conditions, only formic acid is produced (Table 1, entry 3) [42]. The latter stresses the effect of a cation exchange membrane in providing a controlled proton supply to the catholyte, minimizing cathodic formation of formic acid. In these electrosynthesis setups, in which protons are generated at the anode, cathodes with high hydrogen overpotential, like mercury and lead, are necessary to minimize current loss through hydrogen gas formation [41–42].

The counter ion seems to have a significant influence on the fate of the cathodically formed CO_2_^•−^ radical anion. For carboxylation of a carbon skeleton to occur, this radical anion needs to react via the radical centered at its carbon atom. Grinberg et al. claim that metal or ammonium cations can cause a reversible migration of the negative charge between the carbon and oxygen atoms, allowing the CO_2_^•−^ radical anion to react with its C-centered radical [42]. When this CO_2_^•−^ radical anion abstracts a proton from the solvent, proton migration from oxygen to carbon results in formation of a strong covalent CH bond, yielding a formyloxy radical, which is further reduced to a formate anion (Table 1, entry 3). In general terms, one can conclude that the electrocarboxylation of 1,3-butadiene is not efficiently performed in aqueous or protic media. MgO has been considered as an alternative cation source (Table 1, entry 4), but its solubility in organic solvents is low. Using ammonia as proton scavenger, with formation of ammonium counter cations, allows electrocarboxylation of 1,3-butadiene, and can increase the selectivity for the C6 product (Table 1, entries 5 and 6). The faradaic efficiencies, however, are rather low, hence, there has been a search to find alternative reducing agents.

Tetraethylammonium oxalate and formate salts appeared to be very promising for this purpose, fulfilling both the role of electrolyte and reducing agent. Tetraethylammonium cations have high reduction stability, while still possessing good ion pairing properties. The degree of delocalization of the positive charge is large enough to prevent cathodic reduction and small enough to allow a quick and stable interaction with the cathodically formed carboxylate anions. Furthermore, oxalate and formate are easily oxidized at a Pt anode, gradually releasing the tetraethylammonium cations. The combination of both salts in acetonitrile gives near quantitative yields of the C5:C6:C10 product mixture, with C6 as the main product (Table 1, entry 7) [44]. The anodic reaction produces CO_2_ which can directly be used as reactant at the cathode (Scheme 8), sustaining the atom economy of the process. This way, however, there is no net incorporation of gaseous CO_2_ into the organic substrate, but only net conversion of more energetic oxalate and formate. On top of that, it is important to realize that both the MgO and the tetraalkylammonium salts can also be considered as sacrificial reducing agents. Their advantage over sacrificial anodes, however, is an easier implementation in a continuous process. The anodic oxidation of formate generates one CO_2_ molecule and one proton, giving a controlled supply of protons to the cathode (Scheme 8). Conducting the electrocarboxylation with tetraethylammonium oxalate, without formate salts, increases the amount of C6 compared to C5 and C10 [44]. The use of acetonitrile as solvent is ideal when working in an undivided cell, thanks to its adequate oxidation stability and high dielectric constant. Anhydrous conditions and the easier oxidation of oxalate and formate, at a Pt anode, compared to butadiene and the cathodically generated carboxylates, make the use of a membrane redundant, enabling the use of a simple undivided cell [44]. Moreover, the use of membranes in non-aqueous or aprotic environments is unsatisfactory as they become poorly conducting [44]. This procedure was patented and extended to various other substrates, like activated olefins, imines, carbonyl and halogen compounds, and other anions like an azide, which forms inert N_2_ upon oxidation [52].

Eventually the electrocarboxylation of 1,3-butadiene was also conducted with sacrificial anodes, resulting in a high selectivity for the C6 product (Table 1, entries 8 and 9) [48–49]. Working under anhydrous conditions allows using another range of cathode materials, such as nickel, without being limited by the hydrogen overvoltage. The cathode metal has a large effect on the product distribution, acting as a catalyst [44,49]. Secondly, the absence of a membrane allowed the use of high CO_2_ pressures, pushing the selectivity towards the C6 product, completely eliminating C10 formation [49]. The above-mentioned benefits of sacrificial anode systems have lead to the electrocarboxylation of various other conjugated dienes [49–51,53–54]. In order to increase the selectivity for the C6 product, dissolved nickel and iron redox mediators have been used [48,53–54]. The need for such organometallic complexes could be eliminated by direct electrocarboxylation on nickel or stainless steel cathode surfaces [49–50]. Substrates with an internal conjugated system appear to be less reactive towards CO_2_ fixation, due to steric hindrance and the presence of electron donating substituents [49,51]. However, working at atmospheric CO_2_ pressures and at lower current densities allows effectively performing the double carboxylation of internal conjugated double bonds in open chains. This way, conjugated linoleic acids could be dicarboxylated with a yield approaching 80% at current efficiencies of over 50%, opening the reactant scope to other renewable dienes [50]. The occurrence of mechanism I (Scheme 7) was illustrated via the formation of oxalic acid, the CO_2_ dimerization product [50]. Additional insight in the carboxylation mechanism was gained by comparing the reactivity of 1,3-cyclohexadiene with a mixture of 2,4-hexadiene isomers. The fixed cyclic conformation of 1,3-cyclohexadiene increases its reactivity towards CO_2_ fixation, explained by a higher adsorption strength on the cathode surface. This suggests that dienes undergo carboxylation according to mechanism II while adsorbed on the surface, combined with mechanism I (Scheme 7). Moreover it was shown that diene configuration has a strong stereoelectronic effect on the rate of the dicarboxylation, with the *Z,Z*-configuration being the most reactive one [50].

### Electrocarboxylation of olefins and alkynes

**Scheme 9:**
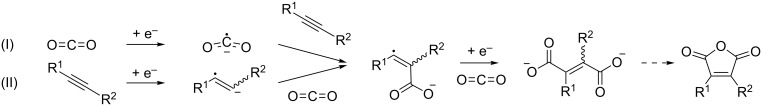
General mechanism for electrocarboxylation of alkynes.

Many reports have been published on the electrocarboxylation of olefins and alkynes [55–74]. Most research has been done using a setup with a sacrificial magnesium or aluminum anode. The general mechanism of alkyne electrocarboxylation to a 1,4-dicarboxylated product is shown in Scheme 9. It is highly similar to the mechanism for electrocarboxylation of olefins. In these reactions a high selectivity for dicarboxylation can be achieved.

**Scheme 10:**
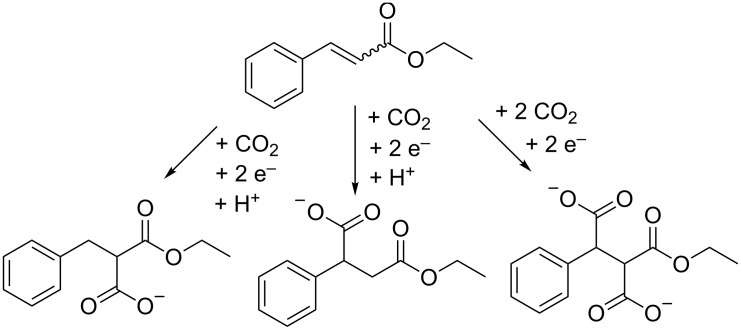
Electrocarboxylation of ethyl cinnamate [70].

Alkynes and olefins react according to similar pathways, although a separate mechanism has been proposed for selective monocarboxylation of alkynes using nickel mediators, usually with bipyridine or *N*,*N*,*N*′,*N*′,*N*′′-pentamethyldiethylenetriamine ligands [55–60]. These organometallic complexes have also proven their value in increasing the carboxylation efficiency of alkenes, without including a specific selectivity for the monocarboxylic acid [56]. Monocarboxylation can readily occur as a side reaction when a small amount of protons are present in the reaction mixture, or through a proton/hydrogen radical abstraction from the reaction medium. The triple bond of alkynes is more active towards carboxylation than the olefin double bond [56]; furthermore, terminal alkynes are more reactive than internal alkynes [56–58], both leading to highly selective CO_2_ fixation. The selectivity towards the dicarboxylation product can be significantly increased by working at higher CO_2_ pressures [61,66], although an optimum must be found to minimize oxalic acid formation at high CO_2_ pressures by electrodimerization of CO_2_ [72]. Under rigorously anhydrous conditions, the dicarboxylation product of alkynes, shown in Scheme 9, can be transformed to a maleic anhydride [61–62]. Alkynes are rather reactive as such; olefins can be rendered more reactive by introduction of electron withdrawing groups [68–71,73–74]. Thus ethyl cinnamate was electrocarboxylated in 78% yield to give a mixture of mono- and dicarboxylated product (Scheme 10) [70]. Activated olefins like dimethyl maleate and acrylonitrile have also been reacted in a setup with a stable anode utilizing tetraethylammonium oxalate, formate or azide salts as the reductant [52].

### Electrocarboxylation of ketones, aldehydes and imines

**Scheme 11:**
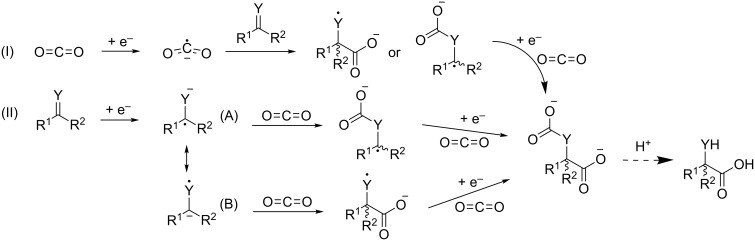
General electrocarboxylation mechanism for carbonyl compounds (Y = O) and imines (Y = NH) [75–77].

As is the case for conjugated dienes, olefins and alkynes, two possible pathways exist for electrocarboxylation of carbonyl and imine compounds. One starts with CO_2_ reduction; another starts with reduction of the substrate (Scheme 11). The second route is considered as the predominant one [75]. In case the carbonyl or imine compound is reduced first, the negative charge may reside either on the carbon or on the heteroatom. This results in a first CO_2_ fixation on the carbon or on the heteroatom, depending on the electron-withdrawing/electron-donating properties of the substituents R^1^ and R^2^. In both cases, a second electron reduction in presence of CO_2_ yields a carboxylate intermediate with an additional carbonate or carbamate group. The latter is converted to the corresponding α-hydroxy acid or to an α-amino acid after acid hydrolysis in the product work-up [75–77].

**Scheme 12:**
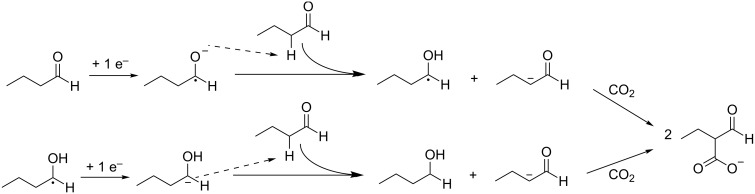
Electrocarboxylation mechanism of butyraldehyde proposed by Doherty [78].

An alternative mechanism has been proposed for aliphatic aldehydes, in which not α-hydroxy acids are formed but in which CO_2_ is incorporated on the α-carbon according to Scheme 12 [78]. Here, the reduced aldehyde abstracts a proton from the α-carbon of an unreacted aldehyde.

The electrocarboxylation of ketones was first described by Wawzonek, converting benzophenone and acetophenone to benzylic acid and 2-hydroxy-2-phenylpropionic acid, respectively [79]. This offers an electrochemical route for several commercially relevant α-aryl propionic acids, used as non-steroidal anti-inflammatory drugs (NSAIDs) [80]. Therefore, the electrocarboxylation of aromatic ketones with sacrificial anodes has been extensively investigated [75–77,81–98]. Some researchers focused on replacing toxic and volatile organic solvents with ionic liquids [81–83]. Their negligible vapor pressure, large electrochemical window, good intrinsic conductivity and high CO_2_ solubility make them interesting solvents for electrochemical CO_2_ valorization [81–83,99–101]. Under similar conditions, ketones are carboxylated with higher selectivity for the α-hydroxy acid compared to aldehydes, and especially compared to aliphatic aldehydes, like acetaldehyde, which give very poor yields [84]. Since carbonyl compounds have the tendency to accept an electron more easily than CO_2_, the reaction mixture contains a lot of carbonyl radical anions, which can form vicinal diol dimers (pinacols) as side product [75–76,83]. The ratio of CO_2_ to substrate is of great importance in obtaining a high selectivity for the α-hydroxy acid products. Working at high CO_2_ pressures and low carbonyl concentrations gives the highest faradaic efficiencies, minimizing pinacol formation. The presence of protons drastically increases the amount of dimerization product and favors the hydrogenation of the carbonyl group to the alcohol [75–76,82,91]. The cathode material again plays an important role in the electrocarboxylation of carbonyl compounds. Toxic lead cathodes [85] and expensive platinum cathodes [87] can easily be replaced by better performing stainless steel [90] and nickel [75,89]. Concerning the reactivity of carbonyl compounds, it has been demonstrated that the carboxylation rate of benzophenones is decreased by electron donating substituents [90]. The pharmaceutical value of the electrocarboxylation of aromatic ketones to NSAIDs has lead to a significant number of patents using a sacrificial anode [92–95]. Since enantioselectivity is crucial for anti-inflammatory drugs, research has also been performed on the enantioselective electrocarboxylation of aromatic ketones, using chiral alkaloids [96–98].

**Scheme 13:**
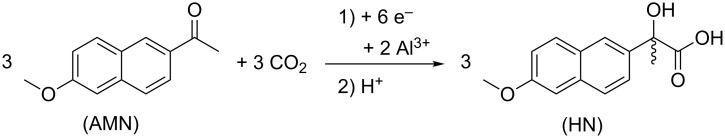
Electrocarboxylation of AMN to HN using a sacrificial aluminum anode [86].

The most important semi-industrial scale electrocarboxylation processes are related to the synthesis of these NSAIDs. The α-hydroxy acid is an intermediate, still requiring a chemical hydrogenation to obtain the desired product. 2-Acetyl-6-methoxynaphthalene (AMN) can be converted to hydroxynaproxen (HN), a precursor of naproxen (Scheme 13) [85–86]. Although yields up to 90% were obtained in a 1 L flow reactor, the switch to a 75 L system was accompanied by leaks and instrument problems resulting in a low yield (58%) and current efficiency (30%) [86].

**Scheme 14:**
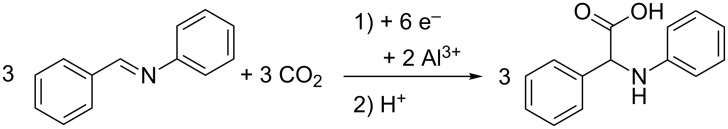
Electrocarboxylation of benzalaniline using a sacrificial aluminum anode [105].

Besides carbonyl compounds, imines have also shown value as substrates for electrocarboxylation, namely in the synthesis of non-natural amino acids [102–108]. A semi-industrial setup was designed for the electrocarboxylation of benzalaniline (Scheme 14). Scale up was done in a filter press type cell with flow distribution, which is commercially available. The electrodes are pressed together with a PTFE coated glass fibre net between them. This way, the inter-electrode gap remains constant during consumption of the anode. In a 2 L solution with 200 g of reactant, a product yield of 85% and a current efficiency of 80% were obtained [105].

**Scheme 15:**
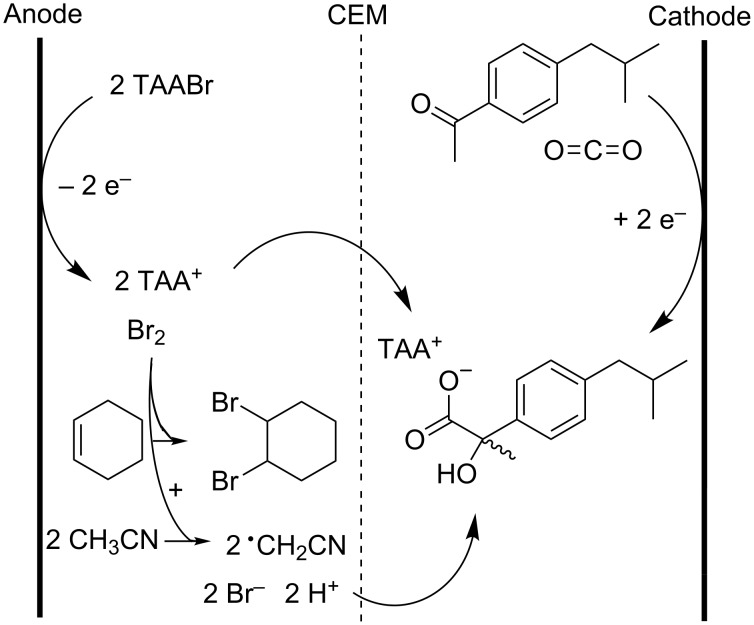
Electrocarboxylation of *p*-isobutylacetophenone with stable electrodes [94–95].

The electrocarboxylation of aromatic ketones was also conducted with stable electrodes as shown in Scheme 15 [94–95]. In these patents *p*-isobutylacetophenone is carboxylated to hydroxyibuprofen, which is readily hydrogenolyzed to ibuprofen. A nafion membrane is used, allowing a selective passage of protons and tetraalkylammonium cations from the anolyte to the catholyte. Cyclohexene is added to the anolyte to scavenge the anodically formed bromine. A current efficiency of up to 90% was reached with a copper cathode and graphite anode. The method appeared also suitable for the synthesis of other NSAIDs like naproxen, cicloprofen, isoprofen, flurbiprofen, fenoprofen and carprofen.

**Scheme 16:**
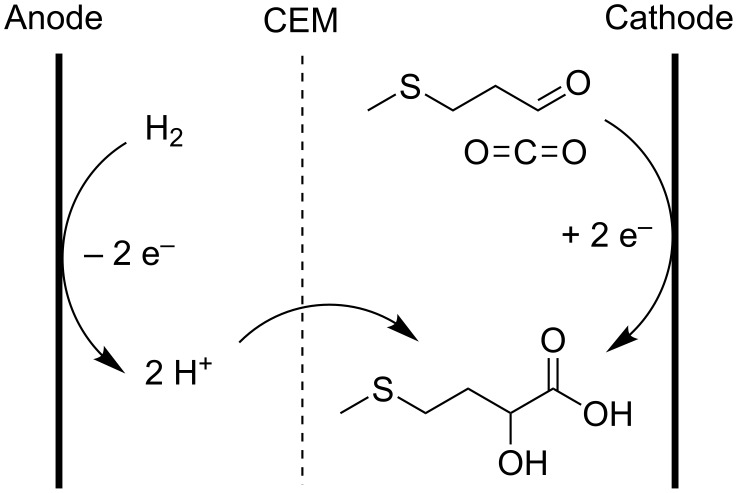
Electrochemical carboxylation of MMP to MHA [110–111].

The electrocarboxylation of aliphatic aldehydes was also patented, namely for the production of 2-hydroxy-4-methylmercaptobutyric acid (MHA) by electrochemical carboxylation of 3-methylmercaptopropionaldehyde (MMP), with both a sacrificial anode [109] and a stable anode [110–111] (Scheme 16). The system, using the stable electrodes, can be extended to aldehydes, ketones and imines.

MHA is an industrial scale feed additive, which is conventionally prepared using cyanides. In the proposed setup for the electrocatalytic preparation of MHA, a boron-doped diamond coated permeable cathode and Pt coated permeable anode rest directly on the cation exchange membrane to decrease ohmic resistance by the membrane, minimizing the required voltage. Hydrogen, supplied to the anolyte gives protons, which gradually enter the catholyte to form the free carboxylic acids. Both anolyte and catholyte are based on DMF. Unfortunately, no yields or current efficiencies higher than 30% were obtained [111].

There are also examples in which aromatic ketones and imines are electrocarboxylated in an undivided cell with stable anode. CO_2_ fixation in acetophenone was done in good yields in an undivided cell using a quaternary ammonium oxalate as an electrolyte and a sacrificial reducing agent [52,94–95,112–113]. Benzalanilines were carboxylated electrochemically with 79% faradaic yield, using an oxalate electrolyte [52].

### Electrocarboxylation of organic halides

**Scheme 17:**
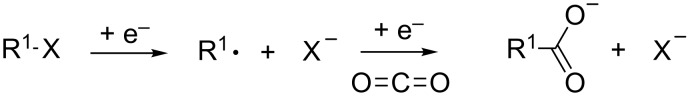
General mechanism for electrocarboxylation of alkyl halides [122,124–126,128].

Numerous reports have been published on the electrocarboxylation of organic halides [114–146]. In a first step, a one electron reduction causes a halide anion to dissociate, forming a reactive radical. The latter undergoes a second reduction in the presence of CO_2_, yielding a monocarboxylate anion (Scheme 17) [122,124–126,128].

The first reactions reported were conducted in a divided cell, giving only moderate yields [114–115]. A drastic increase in efficiency was obtained by employing sacrificial anodes [116], especially magnesium anodes [117–118]. The cathode material is again of great importance, with silver and platinum giving the highest carboxylation selectivity [119–125]. Redox mediators allow working at a less negative cathodic potential, which results in a better energy efficiency and a more selective CO_2_ fixation [128]. The most common redox mediators are organometallic nickel [129–131], palladium [132] and cobalt complexes [133–137].

**Scheme 18:**
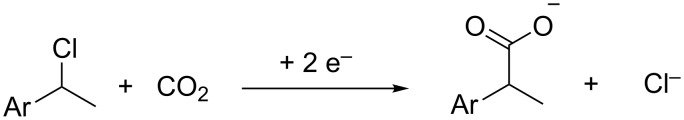
Electrocarboxylation of benzylic chlorides as synthesis route for NSAIDs.

Similar to what was mentioned above for aromatic ketones, benzylic chlorides can also be converted to 2-arylpropionic acids (Scheme 18), with applications in the pharmaceutical industry, mainly as NSAIDs. Here too, some articles described the use of ionic liquids as solvent for electrocarboxylation reactions, in order to increase the safety and efficiency of the process [120,122].

The industrial potential of this reaction has been assessed in several pilot applications, for example, using a setup with a sacrificial anode. In a 400 L reactor, a narrow and constant interelectrode gap was maintained in time and space, avoiding an ohmic drop during consumption of the anode material. A polyethylene grid is placed between the electrodes, allowing the anode to press down on the cathode by its own weight. A good agitation is obtained by pumping the reaction mixture through the cell [138]. Unfortunately, industrial production was not developed because of difficulties in the purification of the product, arising from the presence of impurities generated by the degradation of the solvent [139]. Another pilot scale experiment, conducted for the production of NSAIDs, uses a stable graphite anode in an undivided cell. The anodic reaction is the oxidation of lithium oxalate, giving yields up to 85% of 2-phenylpropionic acid [140]. The same group also investigated a setup in which a metal powder, like zinc, is oxidized at the anode, giving similar results [141].

The electrocarboxylation of organic halides can also be considered as an alternative dechlorination pathway for chlorobenzenes [122] and polychloromethanes [142]. While the synthesis of halogenated reagents is rather hazardous, the electrocarboxylation of organic halides is an interesting method to revalue waste products, like for example carbon tetrachloride, a toxic liquid, causing ozone depletion. In an undivided cell and acetonitrile as solvent, tri- and dichloroacetic acid are formed with current efficiencies between 50 and 60%. The exact anodic reaction(s), however, are not really specified. Oxidation of chloride, which itself originates in the CCl_4_ reactant, likely results in partial chlorination of the acetonitrile solvent, releasing protons which in turn cause the formation of chloroform by attacking the cathodically formed carbanions [143].

**Scheme 19:**
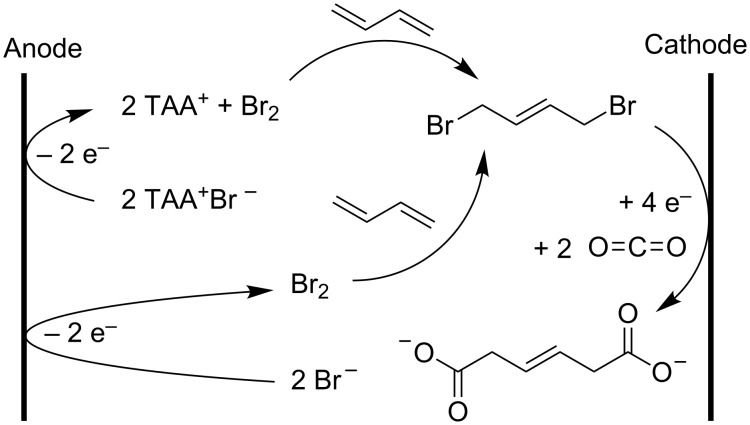
Electrocarboxylation of 1,4-dibromo-2-butene [144].

An undivided electrosynthesis setup with stable anode can also be used for CO_2_ fixation in other aliphatic halides. The anodic oxidation of tetraethylammonium oxalate is used in the electrocarboxylation of 1-bromo-2-methylpentane, which is almost quantitatively converted into 3-methylhexanoic acid [52]. The electrocarboxylation of 1,4-dibromo-2-butene is another reaction for which a stable anode and an undivided cell were proposed. The goal here is to form 3-hexenedioic acid, a precursor of adipic acid, although very poor yields and current efficiencies were obtained. Besides 1,4-dibromo-2-butene, 1,3-butadiene is added in the one-compartment cell to capture the bromine generated at the anode, forming the reactant (Scheme 19). The low yields are caused by debromodimerization and oligomerization [144].

A significant effort has been devoted to efficiently produce cyanoacetic acid through CO_2_ fixation in chloroacetonitrile, as an alternative for the hazardous synthesis via alkali metal cyanides [137,145–146]. Derivatives of cyanoacetic acid are precious starting materials in pharmaceutical and agrochemical synthesis [137]. When the anodic reaction is the oxidation of a halide, lower current efficiencies can be attributed to a successive oxidation and reduction of respectively halides and halonium species. Therefore, the use of a membrane or glass frit can be interesting to minimize this effect. However, in a divided cell, the electrocarboxylation of chloroacetonitrile to cyanoacetic acid still appeared to give higher current efficiencies when using a sacrificial anode [146]. The major downside in carboxylating organic halides is the release of halides in the system, which can moreover induce a significant number of side reactions in a non-sacrificial setup, and which is in any case disadvantageous for the atom economy.

### Innovative electrocarboxylation of other substrates

**Scheme 20:**
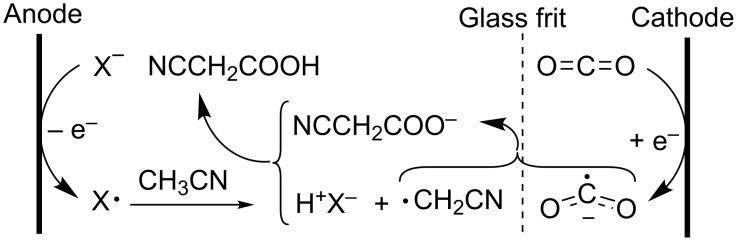
Convergent paired electrosynthesis of cyanoacetic acid, with X^−^ = F_4_B^−^, ClO_4_^−^, HSO_4_^−^, Cl^−^, Br^−^ [147].

Besides the electrocarboxylation of chloroacetonitrile, CO_2_ can also be incorporated electrocatalytically in acetonitrile itself. Such reaction was successfully conducted in a two-compartment cell divided by a medium porosity glass frit (Scheme 20). This is an interesting alternative for the conventional synthesis of cyanoacetic acid, which is carried out by the reaction of chloroacetic acid and alkaline cyanides [147–149].

The electrocarboxylation of acetonitrile to cyanoacetic acid is an example of a convergent paired electrosynthesis, meaning that two different substrates undergo either oxidation or reduction to afford products that react among themselves to generate a single product. Cyanomethyl radicals are formed by anodic oxidation of the supporting electrolyte anion followed by hydrogen radical abstraction from the acetonitrile solvent. These cyanomethyl radicals are then coupled to the CO_2_^•−^ radical anion, forming cyanoacetic acid after protonation. The authors claim that the product is solely formed in the anolyte after CO_2_^•−^ transport from the catholyte to the anolyte, although transport from cyanomethyl radicals to the catholyte is not excluded. However, product formation through cathodic reduction of acetonitrile is ruled out properly, since no cyanoacetic acid was formed when using a cation-exchange membrane. Since most of the product is present in the anolyte, current yields are rather low (24%). Moreover, the electrolyte anion and the cyanoacetic acid product have similar oxidation potentials. On top of that, some halogenation of the solvent to chloroacetonitrile was observed as a side reaction [147]. This reaction setup was also tested with propionitrile, butyronitrile, benzyl chloride and toluene in the anolyte compartment. Adjacent functional groups weaken C–H bonds, yielding relatively stable radicals, in turn resulting in selective CO_2_ fixation [150].

**Scheme 21:**
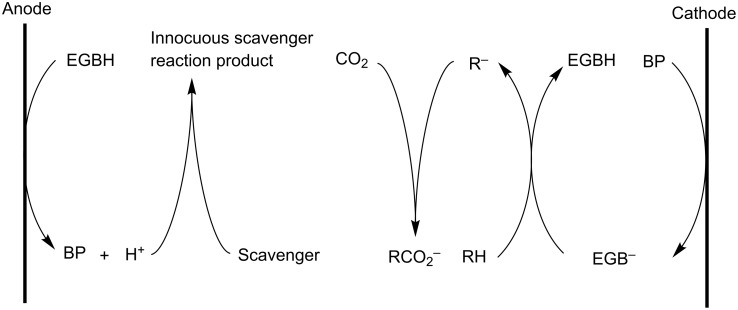
General scheme of carboxylation of weak acidic hydrocarbons with electrogenerated bases. RH: weakly acidic hydrocarbon; BP: base precursor; EGB^−^: electrogenerated base; EGBH: conjugate acid of electrogenerated base [151].

Another patented system uses electrogenerated bases to deprotonate a weakly acidic hydrocarbon group forming anions which are carboxylated in the presence of CO_2_. Meanwhile, proton scavengers remove protons released from the anodic regeneration of the base precursors as shown in Scheme 21 [151].

The electrogenerated bases are redox mediators, used as catalysts in the carboxylation process. The net reaction can be written as follows:

RH + CO_2_ + scavenger → RCOO^−^ + innocuous scavenger reaction product.

**Scheme 22:**
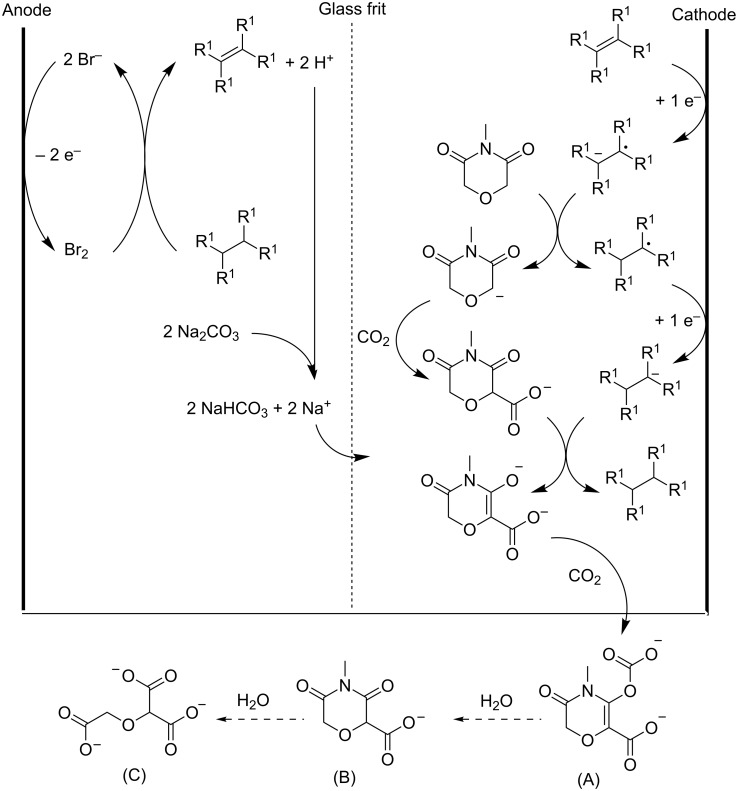
Electrocarboxylation of *N*-methyldiglycolimide to methoxymethane-1,1,1’-tricarboxylate precursors. R^1^: ethoxycarbonyl [151].

The base precursor should be more easily electroreduced than the weakly acidic hydrocarbon group and carbon dioxide, and should not undergo a nucleophilic attack by either the hydrocarbon anion or the electrogenerated base. Therefore, the base precursor should be sterically hindered at or near the site(s) where reduction will occur. The electrogenerated base must be a strong enough Brønsted base to deprotonate the weakly acidic hydrocarbon group. Ethenetetracarboxylate tetraesters are typical base precursors, suited for the electrocarboxylation of *N*-alkyldiglycolimides (Scheme 22). This process provides a feasible route to methoxymethane-1,1,1’-tricarboxylate salts, which are excellent detergent builders. The reaction should be carried out in strictly anhydrous conditions, since water is a stronger acid than the weakly acidic hydrocarbons employed herein. Electrogenerated bromine is used to regenerate the base precursor. Via a radical bromination, followed by a nucleophilic elimination under alkaline conditions, the alkane is oxidized to the alkene base precursor. These alkaline conditions and the four electron withdrawing ethoxy carbonyl groups (R^1^), prevent further bromination of the acquired double bond. Sodium carbonate is a suitable proton scavenger, providing the required alkalinity and being a convenient source of sodium ions. Crown ethers are added to dissolve the alkali metal salts in the organic solvent system. After reaction, anolyte and catholyte are filtered and base precursor and conjugate acid of electrogenerated base must be transferred to the other compartment. In this divided cell a yield of 85% for product (A) could be obtained (Scheme 22). In an undivided setup however, lower yields and current efficiencies were observed [151].

**Scheme 23:**
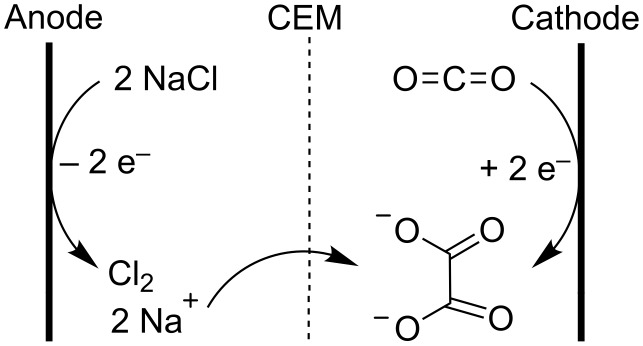
Electrochemical dimerization of CO_2_ with stable electrodes [153].

Oxalic acid is another carboxylic acid which can be formed through electrocarboxylation, namely of CO_2_ itself. This complexing agent has applications in cleaning industry, dyeing processes and metallurgy [152]. Besides its easy synthesis under anhydrous conditions in a cell with sacrificial anode, it can also be produced in a stable electrode setup, with current efficiencies over 50% (Scheme 23) [153]. In the catholyte an organic solvent is used while the anolyte consists of an aqueous NaCl solution. The anodically formed chlorine gas is continuously removed from the anolyte. A cation exchange membrane allows the selective transport of sodium cations to the catholyte. The sodium oxalate that is produced precipitates from the solution. A downside of this setup is the gradual transfer of water from the aqueous anolyte to the organic catholyte, this way steadily lowering the selectivity of the process [153].

## Conclusion

Electrochemical reduction is an efficient approach to activate thermodynamically stable CO_2_ under relatively mild and safe conditions. Electrocarboxylation allows the production of valuable carboxylic acids, through incorporation of CO_2_ in a wide range of organic chemicals. This way, polymer building blocks are produced from conjugated dienes, NSAIDs can easily be obtained from aromatic ketones and benzylic halides, and various other interesting applications are possible. Despite the vast amount of papers and patents on this subject, no industrial applications have emerged yet; only a couple of pilot plant scale processes have been demonstrated. The sustainable and efficient formation of carboxylic acids from carbon dioxide presents many intriguing challenges. The choice of reactor setup, electrode type and reaction pathway, not only affects the implementation cost but also determines operational characteristics like process continuity, atom economy and current efficiency. The shortcomings illustrated in this review emphasize the need for more innovative pathways to invent even more efficient and sustainable electrocarboxylation reactions.
